# Characterization of the Interaction and Cross-Regulation of Three *Mycobacterium tuberculosis* RelBE Modules

**DOI:** 10.1371/journal.pone.0010672

**Published:** 2010-05-17

**Authors:** Min Yang, Chunhui Gao, Yi Wang, Hua Zhang, Zheng-Guo He

**Affiliations:** National Key Laboratory of Agricultural Microbiology, Center for Proteomics Research, College of Life Science and Technology, Huazhong Agricultural University, Wuhan, China; University of Wisconsin-Milwaukee, United States of America

## Abstract

RelBE represents a typical bacterial toxin-antitoxin (TA) system. *Mycobacterium tuberculosis* H37Rv, the pathogen responsible for human tuberculosis, contains three RelBE-like modules, RelBE, RelFG, and RelJK, which are at least partly expressed in human macrophages during infection. RelBE modules appear to be autoregulated in an atypical manner compared to other TA systems; however, the molecular mechanisms and potential interactions between different RelBE modules remain to be elucidated. In the present study, we characterized the interaction and cross-regulation of these Rel toxin-antitoxin modules from this unique pathogen. The physical interactions between the three pairs of RelBE proteins were confirmed and the DNA-binding domain recognized by three RelBE-like pairs and domain structure characteristics were described. The three RelE-like proteins physically interacted with the same RelB-like protein, and could conditionally regulate its binding with promoter DNA. The RelBE-like modules exerted complex cross-regulation effects on mycobacterial growth. The *relB* antitoxin gene could replace *relF* in cross-neutralizing the *relG* toxin gene. Conversely, *relF* enhanced the toxicity of the *relE* toxin gene, while *relB* increased the toxicity of *relK*. This is the first report of interactions between different pairs of RelBE modules of *M. tuberculosis*.

## Introduction

*Mycobacterium tuberculosis*, the causative agent of tuberculosis (TB), continues to pose a serious threat to human health [1]. The persistence, dormancy, and multidrug resistance of this organism and its current co-infection with human immunodeficiency virus (HIV) now make global tuberculosis control particularly challenging [2]. Eradication of tuberculosis is also hampered by our poor understanding of the strategies used by this pathogen for surviving in a dormant state within the phagosome following infection of macrophages [3].

As in a number of other pathogens, dormant infection of *M. tuberculosis* is likely to involve bacterial toxin-antitoxin (TA) systems, which are ubiquitous in free-living bacteria and archaea [4]-[6]. TA modules are defined as protein pairs consisting of a toxin and its antitoxin; the antitoxin can bind to the toxin and neutralize its toxic effects [3]. In typical growth conditions, a pair of toxin-antitoxin proteins will exist as a stable complex [7], [8]. However, in response to stressful or unfavorable growth conditions, the antitoxin is often triggered to degrade, which results in liberation of the toxin. The toxin then exerts its deleterious effects on the host cell [7], [8]. *M. tuberculosis* contains more than 38 toxin-antitoxin loci [8], [9].

Of the bacterial TA systems, the RelBE module is one of the best studied [4], [10], [11]. The toxin induces a global inhibition of translation and arrest of cell growth by cleaving mRNA and tmRNA [4], [8], [11]–[14]. In *E. coli*, RelB has been shown to auto-regulate *relBE* transcription by binding to the *relBE* promoter region, whereas the combined toxin-antitoxin complex strongly inhibits *relBE* transcription [15]–[20]. The *M. tuberculosis* genome harbors three pairs of *relBE* loci, Rv1247c-Rv1246c, Rv2865-Rv2866 and Rv3357-Rv3358, designated as RelBE, RelFG, and RelJK, respectively [8], [9], [21].

Although each pair of *M. tuberculosis relBE*-like genes can form an autoregulatory operon, the regulation patterns appear to be different from those described for a typical TA module [21]. Of the three RelBE pairs, only one toxin (RelJ) has been shown to act as a corepressor of expression. The other two (RelB and RelF) act as transcriptional activators [21]. This indicates that unique intracellular pathogens such as *M. tuberculosis* might show more complex regulation of the expression of their *rel* operons. The actual molecular mechanisms remain to be elucidated in this human pathogen. However, over-expression of individual toxin genes induces growth arrest in a related species, *M. smegmatis*. This phenotype is completely reversible by expression of the cognate antitoxin genes, providing an excellent vehicle for studying the regulation of these genes [21].

In the current study, we have characterized the physical interactions between all three RelB/RelE protein pairs, as well as their interactions with each cognate promoter. The binding regions and sequence characteristics for three RelBE proteins were identified. The RelJ-RelK pair was found to have different regulatory characteristics compared to the other two. In addition, cross regulation between different RelBE modules was examined *in vitro* and *in vivo* in *M. smegmatis*. In particular, two RelB-like proteins were observed to interact with all three RelE-like proteins, RelE, RelG, and RelK.

## Results

### Three *M. tuberculosis* RelB-like proteins physically interact with their cognate RelE-like proteins

**Figure 1:**
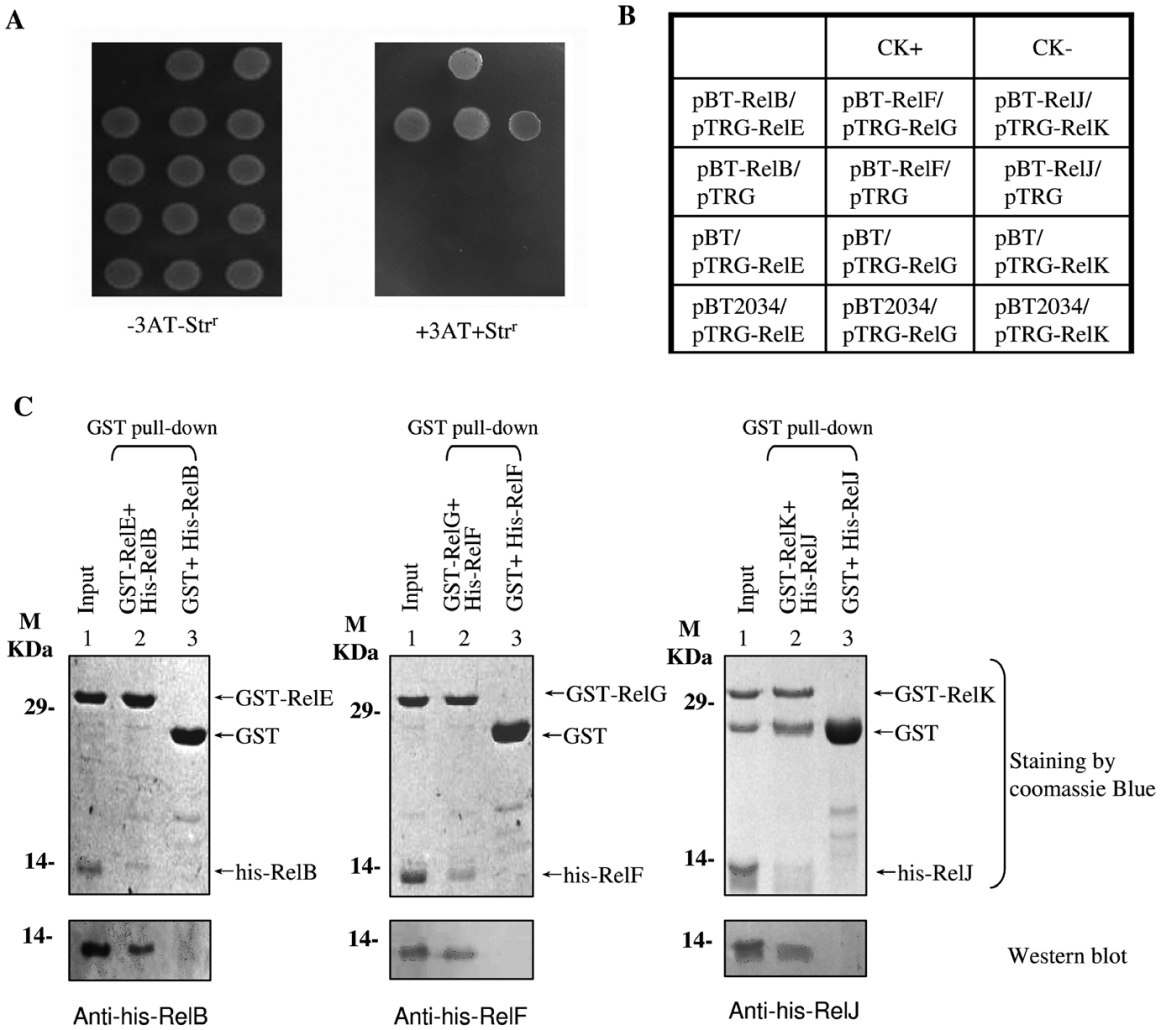
Physical interactions between three pairs of *M. tuberculosis* RelBE-like proteins. (**A**) The BacterioMatch II two-hybrid system (Stratagene) was used to detect protein-protein interactions of RelBE protein pairs, as described in the “Materials and Methods”. Left panel: plate minus streptomycin (str) and 8 mM 3-amino-1, 2, 4-triazole (3-AT). Right panel: plate plus 12 µg/mL str and 8 mM 3AT. (**B**) An outline of the plates in A, CK+: co-transformant containing pBT-LGF2 and pTRG-Gal11P as a positive control. CK-: co-transformant containing pBT and pTRG as a negative control. Each unit represents the corresponding co-transformant in the plates. A non-specific protein, Rv2034, was used as an additional control. (**C**) Pull-down assays for examining the specific interaction between three RelBE protein pairs. The proteins were purified for this assay. Equimolar amounts of 6×His-RelB combined with GST-RelE were used for pull-down assays as described in the “Materials and Methods”. GST was used as the negative control. One predicted size of his-tagged RelB protein, pulled down by their respective cognate GST-tagged RelE protein, was further examined by a Western blotting assay (Fig. 1C, lane 2). “Input” represents a sample removed after the GST-tagged and His-tagged proteins had been combined in the mixture.

The interaction between RelB antitoxin and RelE toxin proteins has been previously characterized [21]. In the present study, we first used a bacterial two-hybrid technique to detect the interactions between the three pairs of *M. tuberculosis* RelBE-like proteins. As shown in Fig. 1A and B, a positive co-transformant (CK^+^) grew on a Screening Medium, but the negative co-transformant (CK^−^) did not grow at all. The co-transformant of RelB/RelE grew well on the screening medium, indicating that the RelB interacted with RelE. Similarly, both RelF/RelG and RelJ/RelK co-transformants grew on the screening medium (Fig. 1A, B), while no growth was observed for their self-activated controls, or for their co-transformants expressing a non-specific protein, Rv2034. Therefore, we were able to successfully detect interactions between all three pairs of RelBE homologs of *M. tuberculosis*.

To confirm these observations, GST pull-down assays were used to characterize the direct physical interactions of the RelBE-like pairs. As shown in Fig. 1C, each pair of RelBE-like proteins was co-purified. One predicted size of his-tagged Rel protein could be readily pulled down by its respective cognate GST-tagged Rel protein, which was clearly demonstrated by a further Western blotting assay (Fig. 1C, lane 2). GST co-incubated with his-tagged Rel proteins did not produce any specific bands (Fig. 1C, lane 3).

### Auto-interaction and conditional cooperativity of three *M. tuberculosis* RelBE-like modules

**Figure 2:**
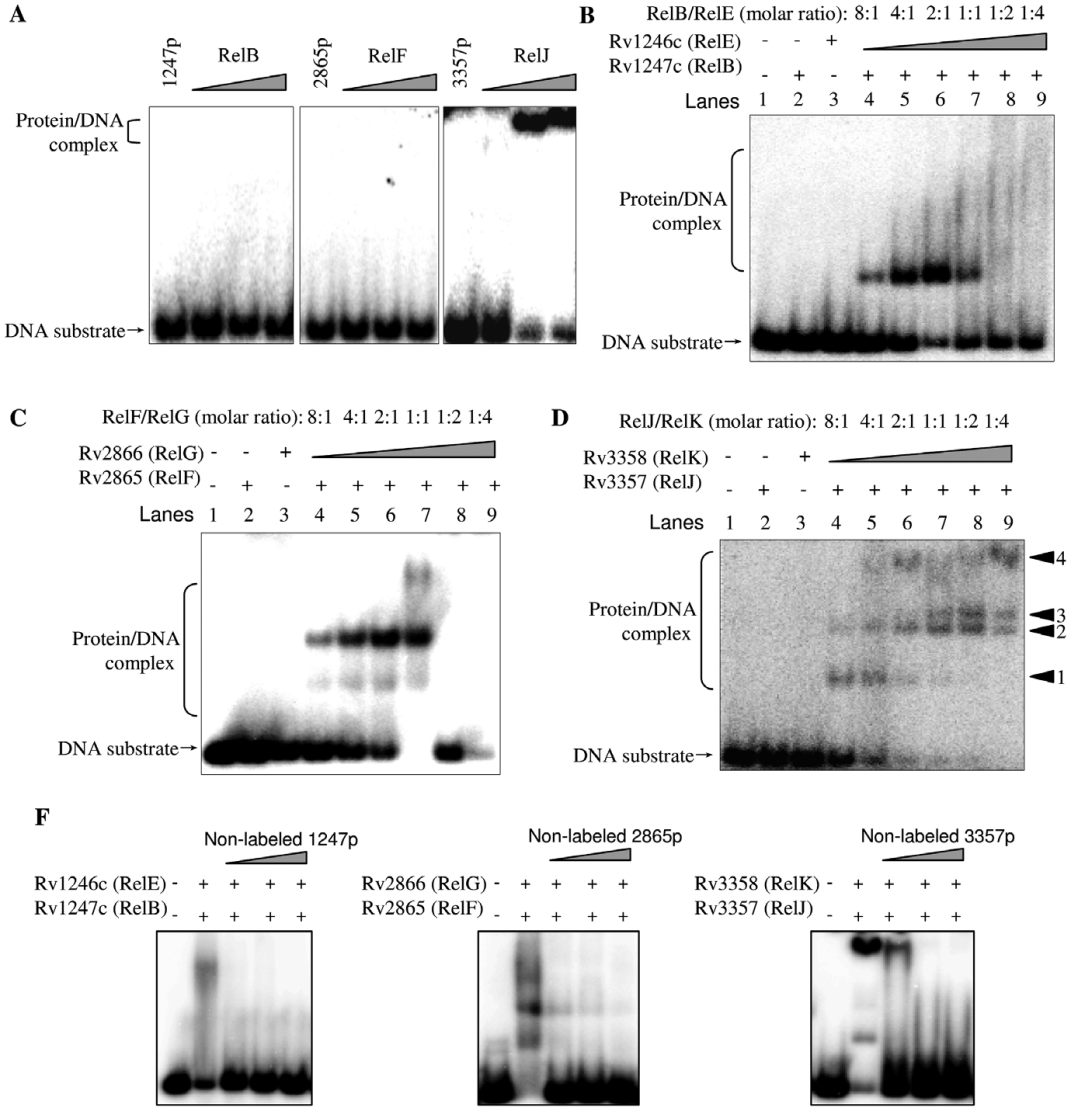
Self-interactions of three *M. tuberculosis* relBE-like modules. Electrophoretic mobility shift assays (EMSA) were used to detect the binding of RelBE proteins with their operon promoters. A fixed amount of ^32^P-labeled DNA substrate was incubated with various amounts of proteins in a total volume of 15 µL of an EMSA buffer. Electrophoresis was performed and gels were exposed to a storage-phosphor screen overnight as described in the “Materials and Methods”. The images were acquired by Typhoon Scanner (GE Healthcare). Both DNA substrate and protein/DNA complexes are indicated by arrows on the left of the figure. (**A**) Different amount of each RelB-like protein (3.75 µM, 7.5 µM, and 15 µM ) interacts with their promoter DNA. (**B**) The concentration of Rv1247c remains constant at 7.5 µM. The interaction between various ratio of RelE/RelB (8∶1, 4∶1, 2∶1, 1∶1, 1∶2, 1∶4) and a fixed amount of DNA substrate. (**C**) The concentration of RelF remains constant at 7.5 µM. The interaction between different ratio of RelF/RelG (8∶1, 4∶1, 2∶1, 1∶1, 1∶2, 1∶4) and a fixed amount of DNA substrate. (**D**) The concentration of RelJ remains constant at 3.75 µM. The interaction between different ratio of RelK/RelJ (8∶1, 4∶1, 2∶1, 1∶1, 1∶2, 1∶4) and a fixed amount of DNA substrate. (**E**) Competitive assays. Three non-labeled promoter DNA substrates (5-fold, 10-fold or 50-fold) were used to compete with their corresponding labeled DNA substrates. The species of promoter DNA was indicated on top of each panel in the figure.

Promoter DNA (described as 1247p, 2865p, and 3357p below) was used as substrate to further investigate the *in vitro* association of RelBE with promoters. As shown in Fig. 2A, of the three RelB-like proteins (from 3.75 to 15 µM), only RelJ associated strongly with its promoter as a singular protein and produced a substantial shifted protein/DNA complex band (Fig. 2A). In contrast, no complex was observed for either the single RelB or RelF antitoxin proteins under similar experimental conditions.

Electrophoretic mobility shift assays (EMSA) were conducted to investigate the effect of the physical interaction between a RelB-like and a RelE-like protein on their promoter-binding abilities. As shown in Fig.2B, neither RelB (7.5 µM) nor RelE (7.5 µM) alone could bind with the promoter. However, as the ratio of RelB/RelE was increased in the reaction mixture (from 8∶1 to 2∶1)(Fig. 2B, lane 4-6), a shifted protein/DNA complex band appeared. The best binding activity was observed when the ratio of RelB/RelE reached 2∶1 (Fig. 2B, lane 6). However, significant inhibition appeared if the ratio was further increased (from 1∶1 to 1∶4) and the protein/DNA complex band disappeared (Fig. 2B, lane 7–9). A similar result was also observed for the interaction between RelF and RelG (Fig. 2C).

As seen in Fig. 2D, neither RelJ alone nor RelK alone formed shifted complexes at a concentration of 3.75 µM (Lane 2 and 3). Four obvious protein/DNA complex bands were observed for the combined RelJ/RelK complex (indicated as band1, 2, 3 and 4 on right of the panel). When the proteins were mixed at a molar ratio of 8∶1 (RelJ still at 3.75 µM), two protein-DNA complex bands were observed (band 1 and 2). As the ratio approached 2∶1, the faster migrating band 1 disappeared while a more slowly migrating band 4 appeared. At a ratio of 1∶1, another band 3 appeared (Lane 7–9). No inhibition was observed with increasing ratios of RelJ/RelK in the reaction mixtures (Fig. 2D, lane 7–9).

To further test the specificity of the interactions between RelBE-like pairs and their promoters, we conducted a competitive experiment. As shown in Fig. 2E, when the labeled DNA substrates remained constant (5 nM), the amount of complex formation between RelBE-like proteins and DNA was significantly decreased and even the bands were disappeared as the concentration of their respective non-labeled promoter DNA substrates (from 25 nM to 250 nM) was raised. This indicated that all three RelBE-like proteins could specifically bind with their promoters.

### Identification of RelBE binding domains within rel promoters

**Figure 3:**
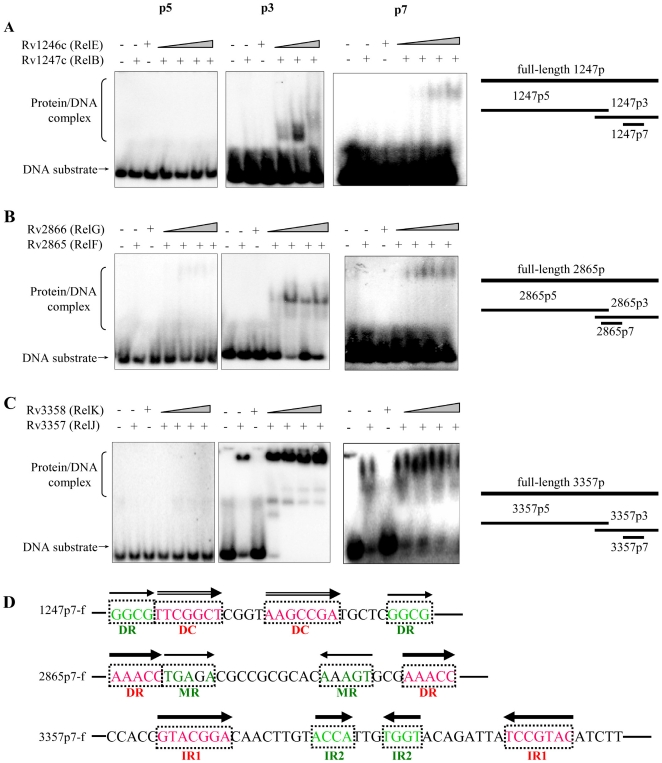
Identification of RelBE binding domains within *rel* promoters. Several short duplex DNA substrates, p5, p3, and p7, were synthesized, which cover different regions within the upstream sequence of each *relBE* operon. These are indicated on the right of the figure. EMSA assays examined the DNA-binding activities of each RelBE pair. The specific DNA substrate was incubated with increasing amounts of RelB-like protein (1.8 µM, 3.6 µM, 7.5 µM, and 15 µM) in a total volume of 15 µL EMSA buffer. 7.5 µM of each RelB-like or RelE-like protein alone was used a control for detecting their respective binding with DNA in each EMSA experiment. Electrophoresis was performed and gels were exposed to a storage-phosphor screen overnight as described in the “Materials and Methods”. DNA substrate and protein/DNA complex were indicated by arrows on the left of the figure. (**A**) The interaction between RelB/RelE and different regions of its operon promoter. (**B**) The interaction between different ratio of RelF/RelG and different regions of its operon promoter. (**C**) The interaction between different ratio of RelJ/RelK and different regions of its operon promoter. (**D**) Structural and sequence characteristics of DNA-binding sites within three relBE operon promoters. IR represents inverted repeat, DR represents direct repeat, DC represents direct complement, and MR represents migrated repeat. All of these sequence motifs are indicated by different arrow types above the corresponding sequences.

The binding sites and target sequence recognized by RelBE-like proteins are unknown in *M. tuberculosis*. The DNA-binding domain for *M. tuberculosis* RelBE proteins was mapped using several short duplex DNA substrates, designated as p5, p3, and p7. These were synthesized to cover different regions within the promoter of each *relBE*-like operon (Fig. 3, right panels; and Table S1 and Table S2). Using these specific DNA substrates, we examined the DNA-binding activities of each RelBE-like pair.

As shown in Fig. 3A, no protein-DNA complex was observed using 1247p5 as a probe (first column). In contrast, a shifted band appeared with stepwise increases in the amount of RelE (from 1.8 to 15 µM) when either 1247p3 (second column) or 1247p7 (third column) were used as DNA substrates. A DNA-binding domain for RelE/RelB was characterized within the 1247p7 fragment (31-bp) (Table S1 and Table S2). Similarly, the domain for RelF/RelG was characterized within the 2865p7 fragment (33-bp) as shown in Fig. 3B. For RelJ/RelK, the DNA-binding activities were observed for either RelJ (7.5 µM) alone or RelJ together with RelK (from 1.8 to 15 µM) on both p3 and p7 DNA substrates (Fig. 3C). These results were consistent with the observations above on the full-length 3357p substrate (Fig. 2D). In contrast, no protein/DNA complex was observed on the p5 substrate (Fig. 3C, first column). Therefore, 3357p7 (50-bp) retained a DNA-binding domain for RelJ/RelK.

When analyzing the binding sequence, we found that many direct repeat, inverted repeat, or direct complement sequence motifs existed within these p7 DNA substrates (Fig. 3D). When analyzed using LOGO software, the three sequences appeared to share some conserved residues, and a consensus sequence of “N(2–3)C∼N∼T∼N(4)C∼N(3) G∼N(4–5) C∼N(2)A∼N(0–1)T∼N(8)” was also established (Figure S1). If half of the conserved 3357p7 sequence boxes was mutated, as shown in Figure S2, the binding ability of RelJK with the DNA obviously decreased. This indicated that the conserved sequence boxes were important for the interaction between Rel proteins and their operons.

### Interactions between three different *M. tuberculosis* RelBE-like modules

**Figure 4:**
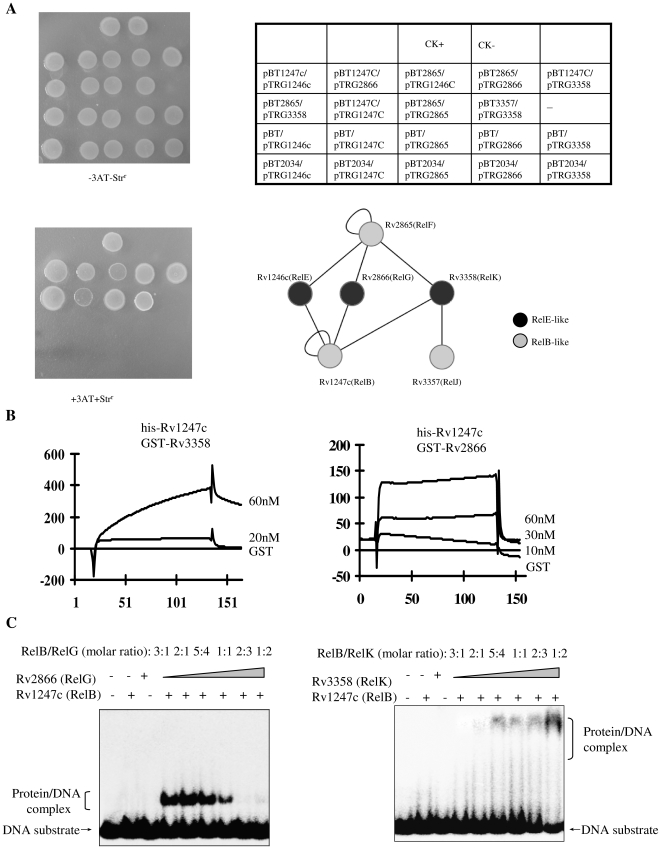
Cross interactions between three pairs of *M. tuberculosis* relBE-like proteins. (**A**) The BacterioMatch II two-hybrid system (Stratagene) was used to detect protein-protein interactions of different pairs of RelBE proteins, as described in the “Materials and Methods”. Upper right panel: an outline of the upper left panel plates (CK+: co-transformant containing pBT-LGF2 and pTRG-Gal11P as a positive control. CK-: co-transformant containing pBT and pTRG as a negative control). Each unit represents the corresponding co-transformant in the plates. Lower left panel: plate plus 10 µg/mL str and 6 mM 3AT. Lower right panel: a summarized network of protein-protein interactions between these RelBE proteins. The black circle represents toxin and the white circle represents antitoxin. (**B**) SPR assays. The interactions of RelB with RelK and RelG were monitored using surface plasmon resonance on a BIAcore 3000 (GE healthcare). The surface of the chip was activated by saturating the nitrilotriaceticacid sites with running buffer (100 mM Hepes-NaOH, pH 75, 50 µM EDTA, 0.1 mM dithiothreitol, 50 mM NaCl) containing 0.5 mM NiCl_2._ In all graphs, time (seconds) is plotted on the X-axis; response units (RU) are plotted on the y axis. Five nmol of histidine-tagged RelB proteins were immobilized on the chip surface. Following a period of stabilization, each GST protein was passed over the chip and then allowed to dissociate for 10 min. Overlay plots depicting the interactions were produced. (**C**) EMSA was used to detect the cross-regulations on the binding of RelBE with Rv1247c operon promoter. Electrophoresis was performed and gels were exposed to a storage-phosphor screen overnight as described in the “Materials and Methods”. Both DNA substrate and protein/DNA complex were indicated by arrows on the left of the figure. 7.5 µM of each of RelB-like and RelE-like protein alone was used a control for detecting their respective binding with DNA. Left panel shows the cross interaction between RelB (7.5 µM) and various concentrations of RelG (2.5 µM, 5 µM, 6 µM, 7.5 µM, 11.25 µM, and 15 µM); right panel shows the interaction between RelB (7.5 µM) and various concentrations of RelK(2.5 µM, 5 µM, 6 µM, 7.5 µM, 11.25 µM, and 15 µM).

To investigate potential communications between different pairs of *M. tuberculosis* RelBE proteins, we assayed the protein-protein interactions among these RelBE-like proteins using bacterial two-hybrid techniques. As shown in Fig. 4A, a group of co-transformants grown on selective medium was successfully isolated (also see Figure S3). A local protein-protein interaction network was constructed based on the screening experiments (Fig. 4A, lower right). Either RelB or RelF antitoxin was able to interact with RelE-like toxin proteins, RelE, RelG, and RelK (Fig. 4A, lower right).

Surface plasmon resonance (SPR) assay confirmed the interaction of RelB with RelG and RelK. As shown in Fig. 4B, his-tagged RelB protein was immobilized on a nitrilotriacetate (NTA) chip. When an increasing amount of GST-tagged RelK protein (20 and 60 nM) was passed over the chip, a significant response of about 400 response units (RU) was observed (Fig. 4B, left panel). Similarly, a response of 140 response units (RU) was observed for the interaction between RelB and RelG (Fig. 4B, right panel). In contrast, no response was observed for GST itself.

Both RelB and RelF physically interacted with all three RelE-like proteins. This suggested that some co-ordinations might exist between different RelBE-like pairs found in *M. tuberculosis*. To test this possibility, we conducted EMSA assays. As shown in Fig. 4C, RelG was capable of a similar regulation of the DNA-binding activity of RelB on its operon promoter (Fig. 4C, left panel) as was seen for RelB with its cognate RelE toxin protein (Fig. 2B). In contrast, the protein/DNA complex shift was slowed when the concentration of RelK toxin protein in the reaction mixture was increased (Fig. 4C, right panel). No binding was observed for RelK alone, even at a high protein concentration. Using a similar EMSA assay, RelE was observed to stimulate the DNA-binding activity of RelF antitoxin protein on its operon promoter (Figure S4A). However, we did not observe the expected effect of RelK on the binding activity of RelF (Figure S4B).

### Cross-regulation of the different *M. tuberculosis* RelBE-like modules

**Figure 5:**
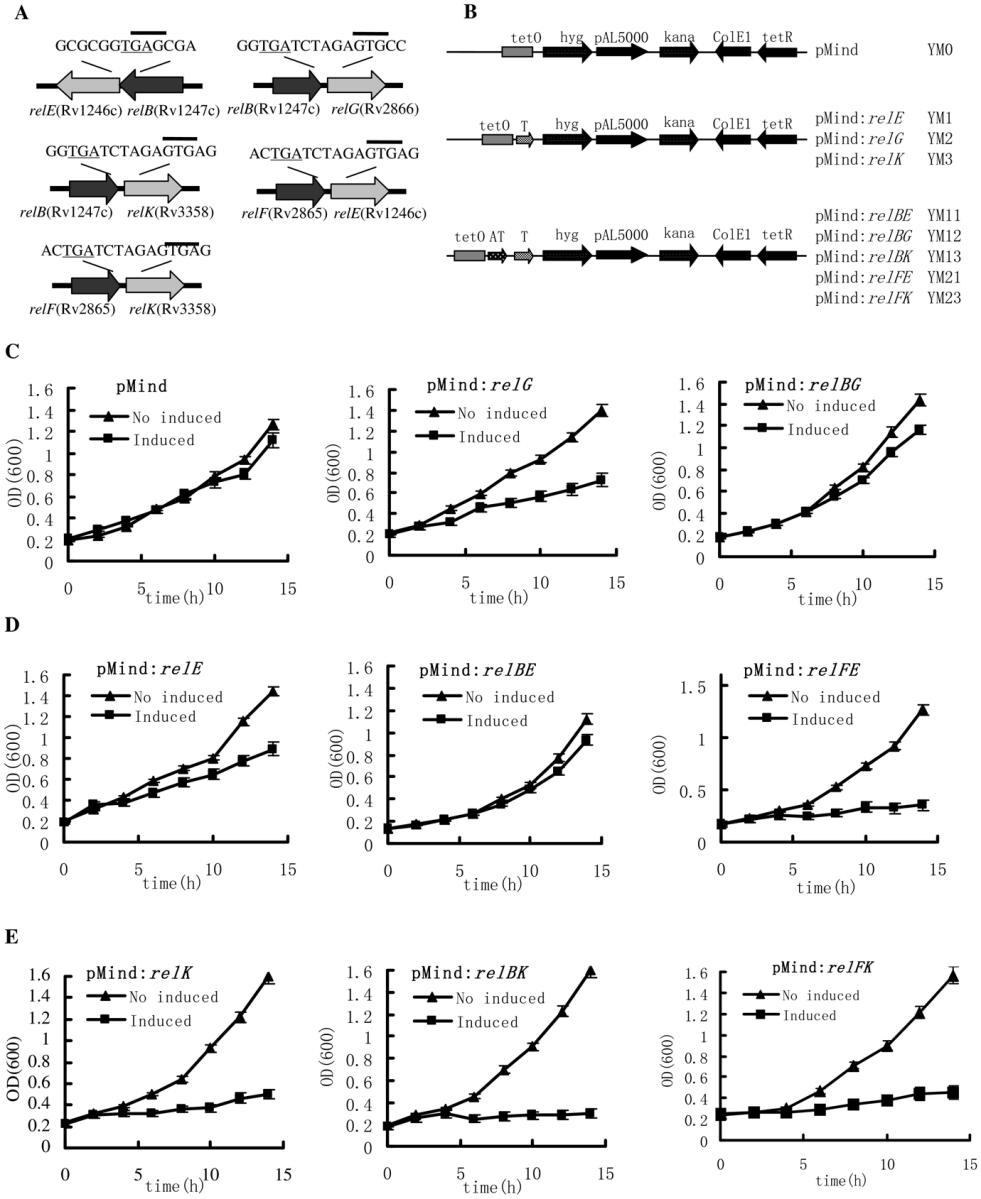
Cross regulation between different *M. tuberculosis* relBE-like proteins on the mycobacterial growth. The single *rel* gene or *relBE*, *relBG*, *relBK*, and *relFE* gene pairs were cloned. A TetR-controlled expression system was used to analyze the effects of *relBE*-like genes on the growth of *M. smegmatis* mc^2^ 155 as described in the “Materials and Methods”. The growth of these recombinant mycobacterial strains were examined in the presence (induction) or absence (no induction) of tetracycline (Tc). Aliquots were taken at the indicated times and the OD_600_ was measured. Each analysis was performed in triplicate. The representative growth curves were plotted. (**A**) Schematic representation of *relBE*, *relBG*, *relBK*, *relFK*, and *relFE*. The GTG start codons are indicated with a line above the codons, and the TGA stop codons are underlined. (**B**) The locations of these genes on the recombinant plasmids. The corresponding strains were demonstrated on the right of the figure panel. (**C**) The effects of single *relG* or *relBG* pair on the growth of *M. smegmatis* mc^2^ 155 in the presence (induction) or absence (no induction) of tetracycline (Tc). (**D**) The effects of single *relE* or *relBE* and *relEF* pairs on the growth of *M. smegmatis* mc^2^ 155. (**E**) The effects of single *relK* or *relBK* and *relFK* pair on the growth of *M. smegmatis* in the presence (induction) or absence (no induction) of Tc.

Cross regulation between different RelBE modules was examined with several mycobacterial growth curves of recombinant *M. smegmatis* strains, with or without induction by tetracycline (Fig. 5). Significant growth inhibition was observed for the recombinant *M. smegmatis* strain containing *relG* alone when induced by tetracycline (Fig. 5C, middle panel). This inhibitory effect was almost eliminated in the *relBG* strain, indicating that the inhibition conferred by *relG* could be rescued by the antitoxin gene *relB* (Fig. 5C, right panel). This effect was consistent with the physical interaction results (Fig. 4C). No growth inhibition was observed for the *M. smegmatis* control strain (Fig. 5C, left panel). Similarly, as shown in Fig.5D (left and middle panels), an inhibitory effect by *relE* and a rescue by *relB* were observed. However, *relF* was unable to rescue the inhibition conferred by *relE*, despite the physical interaction indicated earlier between RelE and RelF (Fig. 4A). Compared to the *relE* toxin gene alone, significantly more inhibition was conferred by *relEF* (Fig. 5D, right panel). No inhibition was observed with the *relF* antitoxin gene expression alone (data not shown). Significant inhibitions were also conferred by *relBK* and *relFK* (Fig. 5E).

**Figure 6:**
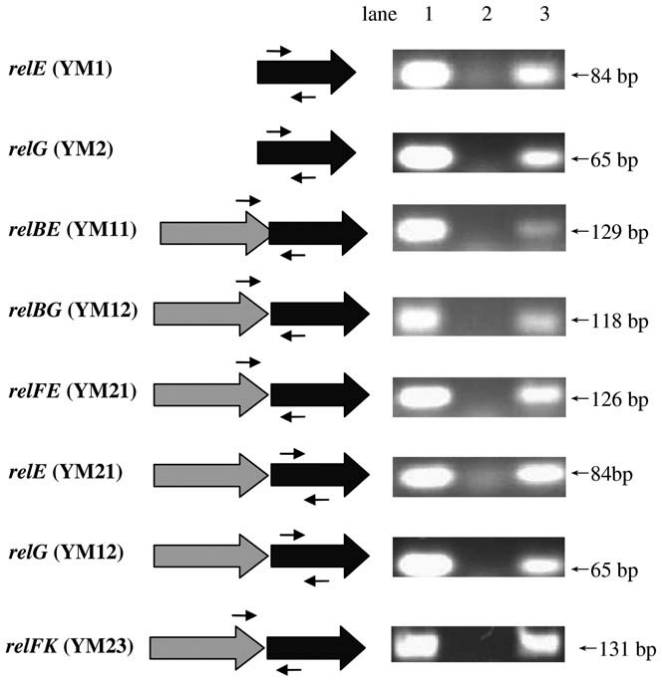
RT-PCR assays for the expressions of *M. tuberculosis* relBE-like proteins in *M. smegmatis* mc^2^ 155. The experiments and assays were performed as described in the “Materials and Methods”. Target genes and their host recombinant strains were demonstrated on the left of panel in the figure. The appropriate sites of each pair of RT-PCR primers were indicated by arrows. The RT-PCR products were detected on 1.5% agarose gel as shown on the right of the panel. Lane 1, positive control (the total DNA of each recombinant strain was used a template for PCR); lane 2, negative control (the cDNA of recombinant strain YM0 was used a template for PCR); lane 3, RT-PCT products (the cDNA of each recombinant strain was used a template for PCR).

To determine if these *rel* genes were expressed at comparable levels in the recombinant *M. smegmatis* strain, specific primers were synthesized (Table S1) and RT-PCR experiments were conducted. As shown in Fig. 6, all *rel* genes or *relBE*-like gene pairs were expressed similarly because the predicted sizes of their cDNA fragments were amplified at similar levels (Fig. 6, right panel).

Therefore, complex cross-regulations were observed between different *M. tuberculosis* RelBE-like modules. The *relB* antitoxin gene could replace *relF* to cross-neutralize the *relG* toxin gene. Conversely, *relF* enhanced the toxicity of the *relE* toxin gene; while *relB* could increase the toxicity of the *relK* toxin gene. *relF* had no obvious effect on the toxicity of *relK*.

## Discussion

Bacterial toxin-antitoxin systems may play crucial roles in controlling dormant infection processes in a number of pathogens [8]. For *M. tuberculosis*, its dormancy within a macrophage could potentially be mediated by three pairs of *relBE*-like genes that are expressed during infection [21]. In the present study, the DNA-binding domain recognized by three RelBE-like pairs and its structural characteristics were revealed. The cross-regulation of the *rel* toxin-antitoxin modules was also confirmed for this unique pathogen.

The three *M. tuberculosis* RelE-like toxin proteins physically interacted with the same RelB-like antitoxin protein. In addition, all three could conditionally regulate the binding of RelB binding with promoter DNA. Complex cross regulating effects of these three RelBE-like modules were also seen on mycobacterial growth in *M. smegmatis*. For example, *relB* could replace *relF* to cross-neutralize the toxin protein *relG*. Conversely, *relF* increased the toxicity of toxin protein *relE,* while *relB* increased that of *relK*. This is the first report of interactions between different pairs of mycobacterial RelBE modules in *M. smegmatis*.

We consistently observed that the RelJ was the only antitoxin that could bind on its own with the promoter region at a relatively low protein concentration (Fig. 2, 3). No binding activity was observed for the other two RelB-like antitoxin proteins (RelB and RelF), even at very high protein concentrations (Fig. 2, 3). However, using a promoter-*lacZ* fusion reporter system, Korch *et al* recently observed that *relB* and *relF* antitoxins on their own could activate the expression of their operons, even though their TA complexes inhibited this expression [21]. One likely explanation is that the binding of RelB or RelF to the promoters might be highly unstable in the absence of complex formation with their respective toxin proteins (RelE and RelG). An *in vitro* assay, such as EMSA, may not be successfully measure this binding as the protein/DNA complex would be unstable. On the other hand, some as yet uncharacterized *in vivo* interactions may also exist for these antitoxins, which might control the expression of their operons. Differential interactions between these toxins and their promoters are likely to be essential for survival of *M. tuberculosis* in a dormant state.

Additional multiple protein/DNA complexes were observed for the interaction of RelJ/RelK with its promoter DNA (Fig. 2D). The DNA-binding activity of the RelJK was constant, even in the presence of increasing amounts of the RelK toxin protein in the reaction mixture. In contrast, the relatively simple protein-DNA bands for RelB/RelE and RelF/RelG showed a conditional cooperativity, as these bands disappeared as the ratio of RelE/RelB-like proteins was increased (Fig. 2B, C). These *in vitro* experiments confirmed the existence of different patterns of interactions between the *M. tuberculosis* RelBE proteins and their operon promoter, which was consistent with the previous *in vivo* transcriptional analysis reported by the Clark-Curtiss group [21]. On the other hand, multiple inverted repeat motifs were found within the characterized binding site for RelJ/RelK, which is similar to the situation described for *E. coli rel* operon modules [19]. However, the sequence motifs within both the RelB/RelE and the RelF/RelG operon modules appeared to have additional complexity, including direct repeat (DR), direct complement (DC), and migrated repeat (MR) sequences. All of these differences and variations in regulation patterns seen for Rel proteins may have communication functions, allowing *M. tuberculosis* to interact with the unique environment within macrophages.

In the current study, we confirmed that cross interactions occur *in vitro* between different *M. tuberculosis* RelBE pairs and *in vivo* in *M. smegmatis*. Construction of a local protein-protein interaction network revealed the unexpected result that the RelB and RelF toxin proteins physically interacted with all three RelE-like toxins. The RelK toxin protein had a similar effect on the DNA-binding activity of RelB to its cognate RelE (Fig. 4C). RelG also stimulated the binding of RelB to the operon promoter. These findings suggest that cross regulation occurs between the different *M. tuberculosis* RelBE-like pairs. The additional finding that the *relB* antitoxin gene could interact with the *relG* toxin gene allows for the possibility that *relB* may replace *relF* to cross-neutralize *relG*. Our *in vivo* growth experiments, which showed that inhibition conferred by *relG* could be rescued by *relB* (Fig. 5C), strongly support this possibility.

Using SCOTS analysis, Korch *et al* recently observed that two *M. tuberculosis* toxins, RelE and RelK, and one antitoxin RelF, were expressed at the later stages of macrophage infection [21], but the biological significance of this was unclear. In the current study, we found that both the RelE and RelK toxins physically interacted with RelF antitoxin (Fig. 4A). In further experiments, RelE toxin was observed to stimulate the DNA-binding activity of RelF antitoxin on its operon promoter (Fig. S4A), while *relF* antitoxin expression enhanced the inhibition of mycobacterial growth by *relE* toxin expression in *M. smegmatis* (Fig. 5D). Interestingly, compared with *relE*, *relK* toxin expression alone strongly inhibited the mycobacterial growth (Fig. 5E, left panel) and the *relF* antitoxin expression did not reduce the inhibition (Fig. 5E, right panel). Our results therefore support a model in which the expression of *relE*, *relF* and *relK* might promote persistence of this pathogen by cooperatively arresting bacterial growth at later stages of macrophage infection [21]. *M. tuberculosis* might use some unique *rel* regulation models to allow it to adapt to the harsh environmental conditions it encounters as it infects a macrophage. Although the exact mechanism of the growth arrest induced by these different RelBE modules remains to be elucidated, the characterization of auto-regulation and cross-regulation presented here provides important information for further research directions.

## Materials and Methods

### Strains, enzymes, plasmids and reagents

*E. coli* BL21 cells and pET28a were purchased from Novagen and used to express *M. tuberculosis* proteins (Table 1 and 2). pBT, pTRG vectors and *E. coli* XR host strains were purchased from Stratagene (Table 1 and 2). Restriction enzymes, T4 ligase, modification enzymes, Pyrobest DNA polymerase, dNTPs and all antibiotics were from TaKaRa Biotech. The reagents for one-hybrid assay and two-hybrid assay were purchased from Stratagene. PCR primers were synthesized by Invitrogen (Table S1). Ni-NTA (Ni^2+^-nitrilotriacetate) agarose was obtained from Qiagen.

**Table 1:** Plasmids used in this study.

Plasmid	genotype or features	Source or reference
pMind	Kan^r^, pAL5000 replicon	5
pMind::relE	relE in BamHI-PacI site of pMind	This study
pMind::relBE	relBE in BamHI-PacI site of pMind	This study
pMind::relBG	relBG in BamHI-PacI site of pMind	This study
pMind::relBK	relBK in BamHI-PacI site of pMind	This study
pMind::relG	relG in BamHI-PacI site of pMind	This study
pMind::relFE	relFE in BamHI-PacI site of pMind	This study
pMind::relK	relK in BamHI-PacI site of pMind	This study
pBT	chlo^r^, p15A replicon, lac-UV5 promoter	Stratagene
pBT-1246c	relE in EcoRI-XbaI sites of pBT	This study
pBT-1247c	relB in EcoRI-XbaI sites of pBT	This study
pBT-2865	relF in EcoRI-NotI sites of pBT	This study
pBT-2866	relG in EcoRI-NotI sites of pBT	This study
pBT-3357	relJ in EcoRI-XbaI sites of pBT	This study
pBT-3358	relK in EcoRI-XbaI sites of pBT	This study
pTRG	tet^r^, ColE1 replicon, lpp/lac-UV5 promoter	Stratagene
pTRG-1246c	relE in EcoRI-XbaI sites of pTRG	This study
pTRG-1247c	relB in EcoRI-XbaI sites of pTRG	This study
pTRG-2865	relF in EcoRI-NotI sites of pTRG	This study
pTRG-2866	relG in EcoRI-NotI sites of pTRG	This study
pTRG-3357	relJ in EcoRI-XbaI sites of pTRG	This study
pTRG-3358	relK in EcoRI-XbaI sites of pTRG	This study
pET28a(+)	Kan^r^, T7 lac promoter, N-terminal His_6_	Novagen
pET-1246c	relE in EcoRI-XbaI sites of pET28a	This study
pET-1247c	relB in EcoRI-XbaI sites of pET28a	This study
pET-2865	relF in EcoRI-NotI sites of pET28a	This study
pET-2866	relG in EcoRI-NotI sites of pET28a	This study
pET-3357	relJ in EcoRI-XbaI sites of pET28a	This study
pET-3358	relK in EcoRI-XbaI sites of pET28a	This study
pGEX	Amp^r^, pBR322 replicon, tac promoter	GE Healthcare
pGEX-1246c	relE in EcoRI-XbaI sites of pGEX	This study
pGEX-1247c	relB in EcoRI-XbaI sites of pGEX	This study
pGEX-2865	relF in EcoRI-NotI sites of pGEX	This study
pGEX-2866	relG in EcoRI-NotI sites of pGEX	This study
pGEX-3357	relJ in EcoRI-XbaI sites of pGEX	This study
pGEX-3358	relK in EcoRI-XbaI sites of pGEX	This study

**Table 2:** Strains used in this study.

Strain or plasmid	Relevant genotype or features	Source or reference
*E.coli*		
DH5a	Host for plasmid construction	TaKaRa
BL21	Host for overexpression	TaKaRa
XR	Host for bacteria two-hybrid()	Stratagene
*M. smegmatis* mc^2^155		30
YM0	mc^2^155with pMind	This study
YM1	mc^2^155with pMind::relE	This study
YM11	mc^2^155with pMind::relBE	This study
YM12	mc^2^155with pMind::relBG	This study
YM13	mc^2^155with pMind::relBK	This study
YM2	mc^2^155with pMind::relG	This study
YM21	mc^2^155with pMind::relFE	This study
YM3	mc^2^155with pMind::relK	This study
YM23	mc^2^155with pMind::relFK	This study

### DNA substrate preparation for DNA-binding activity assays

DNA substrates used in this study include long promoter DNA of three *relBE* modules and their partial fragments. These promoter DNAs, 1247p, 2865p, and 3357p, were amplified by PCR from *M. tuberculosis* H37Rv genomic DNA using their specific primers (Table S1 and Table S2). Short DNA fragments were either amplified by PCR or synthesized directly by Invitrogen (Table S3). The amplified products were purified with the BioFlux PCR DNA Purification kit (BioFlux) and labeled with T4 polynucleotide kinase (Takara) and [γ-^32^P] ATP following the manufacturer's instructions. The mixture was treated at 65°C for 7 min to inactivate the protein kinase in the reactions. The labeled DNA substrates were stored at −20°C until use. The synthesized oligonucleotide was radioactively labeled at its 5′-terminus with T4 polynucleotide kinase (Takara) and [γ-^32^P] ATP. The labeled oligonucleotide was purified as described previously [22], then 1.2 fold unlabelled reverse oligonucleotide was added and incubated at 95°C for 10 min to allow complete annealing. The double DNA substrates were stored at −20°C for use.

### Electrophoretic mobility shift assay (EMSA)

The binding of RelBE proteins to DNA was performed using a modification of an electrophoretic mobility shift assay (EMSA). The reactions (15 µL) contained 5 nM ^32^P-labeled DNA fragments, and various concentrations (from 0.5 to 30 µM) of RelB/RelE or their mixed proteins. The reaction mixtures were incubated at 4°C for 30 min in a total volume of 15 µL of an EMSA buffer consisting of 50 mM Tris-HCl, pH7.5, 10 mM MgCl_2_, 1 mM DTT, and 50 mM NaCl. The mixtures were directly subjected to 5% native PAGE containing 0.5× Tris-borate-EDTA (TBE) buffer. Electrophoresis was performed at 200 V at 4°C or in an ice-bath until the bromophenol blue indicator dye reached the bottom of the gel. Gels were exposed to a storage-phosphor screen overnight at room temperature. The images were acquired by a Typhoon Scanner (GE healthcare).

### Cloning, expression and purification of recombinant proteins

Three *relB* and *relE* genes were amplified by PCR using their specific primer pairs (Table S1 and Table S2) from genomic DNA of *M. tuberculosis*. These genes were cloned into the modified pET28a or pGEX-4T-1 expression vectors to produce recombinant plasmids (Table 1). *E. coli* BL21 cells transformed with the recombinant plasmid was grown in 200 mL of LB medium up to an OD_600_ of 0.6. Protein expression was induced by the addition of 0.3 mM Isopropyl β-D-1-thiogalactopyranoside (IPTG). Harvested cells were resuspended and sonicated in binding buffer (100 mM Tris-HCl, pH 8.0, 500 mM NaCl, and 10 mM imidazole for his-tagged proteins or 1x PBS buffer for GST-tagged proteins) and lysate was centrifuged at 10000×g for 30 min. The cleared supernatant was loaded onto an affinity column and washed with wash buffer (100 mM Tris-HCl, pH 8.0, 500 mM NaCl, and 40 mM imidazole for his-tagged proteins, or PBS buffer for GST-tagged proteins). The protein was then eluted using elution buffer (100 mM Tris-HCl, pH 8.0, 500 mM NaCl, and 250 mM imidazole for his-tagged proteins or PBS buffer containing 10 mM reduced glutathione (GSH) for GST-tagged proteins). The eluate was then dialyzed against the buffer (10mM Tris-HCl 100 mM NaCl 1 mM DTT 10% glycerol) overnight and stored at –80°C. Purity of the proteins was greater than 98% as determined by SDS–PAGE and subsequent staining with Coomassie Blue.

### Bacterial two-hybrid assay

The BacterioMatch II Two-Hybrid System (Stratagene) was used to establish protein–protein interactions between *M. tuberculosis* RelB and RelE proteins as described previously [23], [24]. Three *relB* and *relE* genes were amplified by PCR using their specific primer pairs (Table S1 and Table S2) from genomic DNA of *M. tuberculosis*. After digestion with a pair of restriction enzymes (indicated in Table S1), these gene fragments were cloned into the modified pBT or pTRG to produce recombinant vectors (Table 1). A pair of pBT/pTRG plasmids was co-transformed into the reporter strain and spotted onto screening medium containing 6∼8 mM 3-AT, 10∼12 µg/mL streptomycin, 15 µg/mL tetracycline, 34 µg/mL chloramphenicol, and 50 µg/mL kanamycin. The plates were incubated at 30°C for 3–4 days. A co-transformant containing pBT-LGF2 and pTRG-Gal11P (Stratagene) was used as a positive control for expected growth on the Selective Screening Medium. A co-transformant containing empty vector pBT and pTRG was used as a negative control.

### SPR analysis

Surface Plasmon Resonance (SPR) analysis on a Biacore 3000 instrument (GE healthcare) with NTA sensor chips was performed according to our previous published procedures [22], [24], [25]. Briefly, His-tagged RelB-like or RelE-like protein was immobilized onto the NTA chips (Nitrilotriacetic acid chip). The purified GST RelB-like or RelE-like protein, to be used as the ligand, was diluted in the HBS buffer (10 mM Hepes (pH 7.4), 150 mM NaCl, 50 µM EDTA, 5 mM ATP, 0.005% BIAcore surfactant P20) at a concentration of <200 nM and injected at 10 µl/min for 5 min at 25°C. For a negative control, GST protein was substituted for the GST-RelBE protein. Each analysis was performed in triplicate. An overlay plot was produced using BIAevaluation 3.1 software to depict the interaction between RelBE proteins.

### GST pull-down assay

Equimolar amounts of normalized GST or GST-RelB-like proteins were combined with equimolar amounts of normalized his-tagged-RelE-like proteins in 1.5 mL tubes containing 500 µL of PBS. The protein mixture was gently rocked at 4°C for 4–15 hour. Before further purification, 60 µL of mixture was removed and saved as a loading control. The remaining mixtures were then purified using the GST-affinity assay as described above. All samples were subjected to SDS-PAGE and detected by Coomassie blue staining. Images were then acquired by Gel Doc XR (Bio-Rad).

### Assay for toxin growth inhibition

A TetR-controlled expression system was used to analyze the effects of *relBE*-like genes on the growth of *M. smegmatis* mc^2^ 155 [26]. Three toxin genes-*relE*, *relG*, and *relK*-were cloned separately into pMind [27] and the plasmids pMind∶*relE*, pMind∶*relG* and pMind∶*relK* were produced (Table 1). These recombinant plasmids were then transformed into *M. smegmatis* to generate recombinant strains YM1, YM2, and YM3, respectively (Table 2). The *relBE*, *relBG*, *relBK*, and *relFE* gene pairs were also cloned and transformed into *M. smegmatis* to generate corresponding recombinant strainsYM11, YM12, YM13, YM21 and YM23 respectively (Table 2). The strain YM0, containing the empty pMind plasmid (Table 2), was used as negative control. In all assays, tetracycline was used to induce gene expression [28]. The growth of these recombinant mycobacterial strains were examined in the presence (induction) or absence (no induction) of tetracycline (Tc). Cells were grown at 37°C with aeration in 7H9-Kan-Tw (7H9 medium supplemented with 0.5% Tween 80, 30 µg/mL kanamycin, and 0.2% glycerol). When cells entered into a stationary growth phase with an OD_600_ of 1.5 to 2.0, the cultures were diluted in 7H9-Kan-Tw medium to an OD_600_ of 0.2, with an additional growth at 37°C at 200 rpm for 2 hours, and were split for induction with 20 µg/mL Tc vs no induction. Aliquots were taken at the indicated times, and the OD_600_ was measured. Each analysis was performed in triplicate. The representative growth curves are plotted in the figures.

### RT-PCR assays

RNA was isolated from *M. smegmatis* mc^2^ 155 recombinant strains YM1, YM2, YM11,YM12, YM21, YM23, and YM0 (Table 2), respectively. For Reverse-transcription PCR, RNA of recombinant strains was used as a template for synthesis of cDNA using a ReverTra Ace first-strand cDNA synthesis kit(TOYOBO, JAPAN) and reverse primers (Invitrogen) according to the manufacturer's instructions. The cDNA was used to amplify a product encompassing the some parts of *relE*, *relG*, *relBE*, *relBG*, *relFK*, *relFE* genes using their specific primers (Table S1). For a positive control, the total DNA of each recombinant strain was used as a template to amplify a product. The cDNA of recombinant strain (YM0) was used as a template for a negative control. The PCR products were detected on 1.5% agarose gel.

## Supporting Information

Table S1 Primers used in the construction of recombinant vectors.
(0.07 MB DOC)

Table S2 S2 Primers used for amplifying promoter DNA fragments.
(0.05 MB DOC)

Table S3 DNA substrate fragment synthesized for EMSA assays.
(0.03 MB DOC)

Figure S1 LOGO assays for the consensus sequence of three RelBE-binding sites of M. tuberculosis. Sequence alignment was carried by ClustalW toolkit and visualized by BioEdit software locally. Sequence logo were generated by WebLogo tool version 2.8.2 with some parameter optimized according its manual book.
(0.20 MB DOC)

Figure S2 EMSA assay for comparing the binding of RelK/RelJ with wild-type and mutant substrates. EMSA and electrophoresis assays were performed as described in the “Materials and Methods”. Wild-type and mutant 3357p7 were used to compare the binding of RelK/RelJ with two substrates. 3357p7 mutant substrates contain mutations within the half of the conserved 3357p7 sequence boxes (DR and MR). The reaction mixtures contain a constant concentration of RelJ (5 µM) and various concentrations of RelK (2.5 µM, 5 µM, and 7.5 µM).
(0.13 MB DOC)

Figure S3 Cross interactions between three pairs of relBE-like genes of M. tuberculosis. The BacterioMatch II two-hybrid system (Stratagene) was used to detect protein-protein interactions of these RelBE protein pairs, as described in the “Materials and Methods”. Up left panel: plate minus streptomycin (str) and 6 mM 3-amino-1, 2, 4-triazole (3-AT). Up right panel: plate plus 10 µg/mL str and 6 mM 3AT. Down panel: an outline of the plates in A, CK+: co-transformant containing pBT-LGF2 and pTRG-Gal11P as a positive control. CK-: co-transformant containing pBT and pTRG as a negative control. Each unit represents the corresponding co-transformant in the plates. All recombinant plasmids and their containing genes were indicated.
(0.33 MB DOC)

Figure S4 EMSA was used to detect the cross-regulations on the bindings of RelFE (A) and RelFK (B) with Rv2865 operon promoter. Electrophoresis was performed and gels were exposed to a storage-phosphor screen overnight as described in the “Materials and Methods”. Both DNA substrate and protein/DNA complex were indicated by arrows on the left of the figure.
(0.23 MB DOC)
