# Supplementary material for: Characterization of the Interaction and Cross-Regulation of Three Mycobacterium tuberculosis RelBE Modules
Source: PLoS One. 2010 May 17;5(5):e10672. doi: 10.1371/journal.pone.0010672 (PMC2871789; doi:10.1371/journal.pone.0010672)
Supplement: Table S1 — Primers used in the construction of recombinant vectors. (0.07 MB DOC) [file pone.0010672.s001.doc]

| Name | Sequence 5’–3’ | Enzyme | Usage |
| --- | --- | --- | --- |
| Rv1246cf | AGTCGAATTCTAGTGAGCGACGACCATCCCTA | EcoRI | Clone and expression |
| Rv1246cr | TCTATATCTAGATTAACGTGGCCGGCACGGGT | XbaI | Clone and expression |
| Rv1247cf | AGTCGAATTCTAATGGCTGTTGTCCCACTGGG | EcoRI | Clone and expression |
| Rv1247cr | TCTATATCTAGATCACCGCGCGGTGTAACGGT | XbaI | Clone and expression |
| Rv2865f | ATATGCGGCCGCAATGCGGATACTGCCGATTTC | NotI | Clone and expression |
| Rv2865r | AGATAATTCTAGATCAGTGGGGGCGTCGCGGGA | XbaI | Clone and expression |
| Rv2866f | ATATGCGGCCGCAGTGCCTTACACCGTGCGGTT | NotI | Clone and expression |
| Rv2866r | CGCCACCCTCTAGACTATCGGCGGTAGATGTCC | XbaI | Clone and expression |
| Rv3357f | AGAGGAATTCGCATGAGCATCAGTGCGAGCGA | EcoRI | Clone and expression |
| Rv3357r | ATATTATCTAGATCACTCCTCGCCGCCGGCCA | XbaI | Clone and expression |
| Rv3358f | AGATGAATTCAAGTGAGAAGCGTCAACTTCGA | EcoRI | Clone and expression |
| Rv3358r | AATTAATCTAGATCAGTAGTGGTATCGGGCCT | XbaI | Clone and expression |
| Y2866f | TAGATCTAGAGTGCCTTACACCGTGCGGTT | XbaI | Integrate with *relB* |
| Y2866r | TATAACTCGAGCTATCGGCGGTAGATGTCC | XhoI | Integrate with *relB* |
| Y3358f | CGAGTTCTAGAGTGAGAAGCGTCAACTTCGA | XbaI | Integrate with *relB* |
| Y3358r | TATAACTCGAGTCAGTAGTGGTATCGGGCCT | XhoI | Integrate with *relB* |
| Y1246cf | CCGATCTAGAGTGAGCGACGACCATCCCTA | XbaI | Integrate with *relF* |
| Y1246cr | TATACTCGAGTTAACGTGGCCGGCACGGGT | XhoI | Integrate with *relF* |
| M1247f | TATAATGGATCCATGGCTGTTGTCCCACTGGG | BamHI | Clone to pMind vector |
| M1246cf | TATATAGGGATCCGTGAGCGACGACCATCCCTA | BamHI | Clone to pMind vector |
| M1246cr | GTGCTTAATTAAGTTAACGTGGCCGGCACGGGT | PacI | Clone to pMind vector |
| M2866f | TATAAGGATCCGTGCCTTACACCGTGCGGTT | BamHI | Clone to pMind vector |
| M2866r | GCGCTTAATTAACTATCGGCGGTAGATGTCC | PacI | Clone to pMind vector |
| M3358f | TATATAGGATCCGTGAGAAGCGTCAACTTCGA | BamHI | Clone to pMind vector |
| M3358r | TCGCTTAATTAATCAGTAGTGGTATCGGGCCT | PacI | Clone to pMind vector |
| M2865f | TATTAGGATCCATGCGGATACTGCCGATTTCGA | BamHI | Clone to pMind vector |
| relBE-RTf | AGGGCGCTTCGTGAGCAA |  | Reverse transcription -PCR |
| relBE-RTr | GGCGATCTTTTCGGGTAA |  | Reverse transcription -PCR |
| relBG-RTf | AGGGCGCTTCGTGAGCAA |  | Reverse transcription -PCR |
| relBG-RTr | CGGTGGCAGCTTGTGGAG |  | Reverse transcription -PCR |
| relE-RTf | GCGACGACCATCCCTACCA |  | Reverse transcription -PCR |
| relE-RTr | ATGCGGCGGCGATCTTTT |  | Reverse transcription -PCR |
| relFE-RTf | ACGGCGAAGACGAGATCC |  | Reverse transcription -PCR |
| relFE-RTr | GGGTAAGCGTTGCAGGTC |  | Reverse transcription -PCR |
| relG-RTf | ACCTCCACAAGCTGCCACC |  | Reverse transcription -PCR |
| relG-RTr | CGACAGATCGCCGAACGC |  | Reverse transcription -PCR |
| relFK-RTf | ACGGCGAAGACGAGATCC |  | Reverse transcription -PCR |
| relFK-RTr | TTTTGCGATCAGCGGCCA |  | Reverse transcription -PCR |
