# Supplementary material for: Characterization of the Interaction and Cross-Regulation of Three Mycobacterium tuberculosis RelBE Modules
Source: PLoS One. 2010 May 17;5(5):e10672. doi: 10.1371/journal.pone.0010672 (PMC2871789; doi:10.1371/journal.pone.0010672)
Supplement: Table S2 — S2 Primers used for amplifying promoter DNA fragments. (0.05 MB DOC) [file pone.0010672.s002.doc]

| Name | Sequence 5’–3’ | Enzyme | Usage |
| --- | --- | --- | --- |
| 1247cpf | ACCGTCTAGATGACAAGTTGGCCGGGATCA | *Xba*I | Amplify *relO* fragment 1247p |
| 1247cpr | TATACCCAGTGGGACAACAGCCATAACCGC |  |
| 1247p5f | ACCGTCTAGATGACAAGTTGGCCGGGATCA | *Xba*I | Amplify *relO* fragment 1247p5 |
| 1247p5r | TCCGAACGCCTCGTCGCTGTTT |  |
| 1247p3f | GGCGTTCGGCTCGGTAAGCCGA |  | Amplify *relO* fragment 1247p3 |
| 1247p3r | TATACCCAGTGGGACAACAGCCATAACCGC |  |
| 2865pf | ATCTTCTAGAACACACCGAGACCGAACCGT | *Xba*I | Amplify *relO* fragment 2865p |
| 2865pr | TATAGAAATCGGCAGTATCCGCATACCGCC |  |
| 2865p5f | ATCTTCTAGAACACACCGAGACCGAACCGT | *Xba*I | Amplify *relO* fragment 2865p5 |
| 2865p5r | CGTCTCAGGTTTGCTGGTGCTT |  |
| 2865p3f | CACAAAGTGCGAAACCACTGGA |  | Amplify *relO* fragment 2865p3 |
| 2865p3r | TATAGAAATCGGCAGTATCCGCATACCGCC |  |
| 3357pf | ATCTTCTAGACCCAAGCCGGGCGTGCGGCC | *Xba*I | Amplify *relO* fragment 3357p |
| 3357pr | TATACAGGCGCTGCCTCGCCTCGCTCGCTC |  |
| 3357p5f | ATCTTCTAGACCCAAGCCGGGCGTGCGGCC | *Xba*I | Amplify *relO* fragment 3357p5 |
| 3357p5r | CGGTGGGCGCGTGAGCG |  |
| 3357p3f | GCGCCCACCGTACGGAC |  | Amplify *relO* fragment 3357p3 |
| 3357p3r | CAGGCGCTGCCTCGCCT |  |

Notes: Restriction enzyme sites are underlined.
