# Supplementary material for: Characterization of the Interaction and Cross-Regulation of Three Mycobacterium tuberculosis RelBE Modules
Source: PLoS One. 2010 May 17;5(5):e10672. doi: 10.1371/journal.pone.0010672 (PMC2871789; doi:10.1371/journal.pone.0010672)
Supplement: Table S3 — DNA substrate fragment synthesized for EMSA assays. (0.03 MB DOC) [file pone.0010672.s003.doc]

| Name | Sequence |
| --- | --- |
| 1247p7 | 5’-GGCGTTCGGCTCGGTAAGCCGATGCTCGGCG-3’  3’-CGCCGAGCATCGGCTTACCGAGCCGAACGCC-5’ |
| 2865p7 | 5’-AAACCTGAGACGCCGCGCACAAAGTGCGAAACC-3’  3’-GGTTTCGCACTTTGTGCGCGGCGTCTCAGGTTT-5’ |
| 3357p7 | 5’-CCACCGTACGGACAACTTGTACCATTGTGGTACAGATTATCCGTACATCTT-3’  3’-AAGATGTACGGATAATCTGTACCACAATGGTACAAGTTGTCCGTACGGTGG-5’ |
