# Supplementary figures and images for: Characterization of the Interaction and Cross-Regulation of Three Mycobacterium tuberculosis RelBE Modules
Source: PLoS One. 2010 May 17;5(5):e10672. doi: 10.1371/journal.pone.0010672 (PMC2871789; doi:10.1371/journal.pone.0010672)

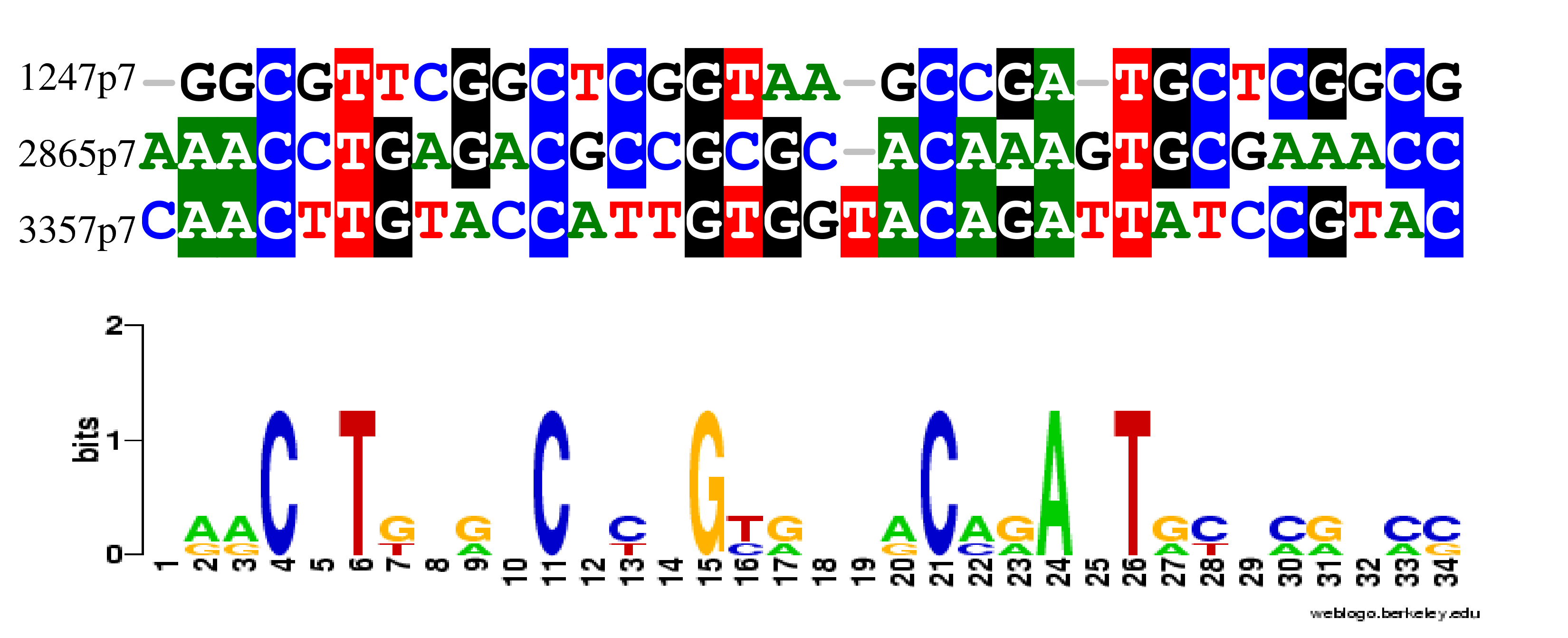

Supplement: Figure S1 — LOGO assays for the consensus sequence of three RelBE-binding sites of M. tuberculosis. Sequence alignment was carried by ClustalW toolkit and visualized by BioEdit software locally. Sequence logo were generated by WebLogo tool version 2.8.2 with some parameter optimized according its manual book. (0.20 MB DOC) [file pone.0010672.s004.doc]

**
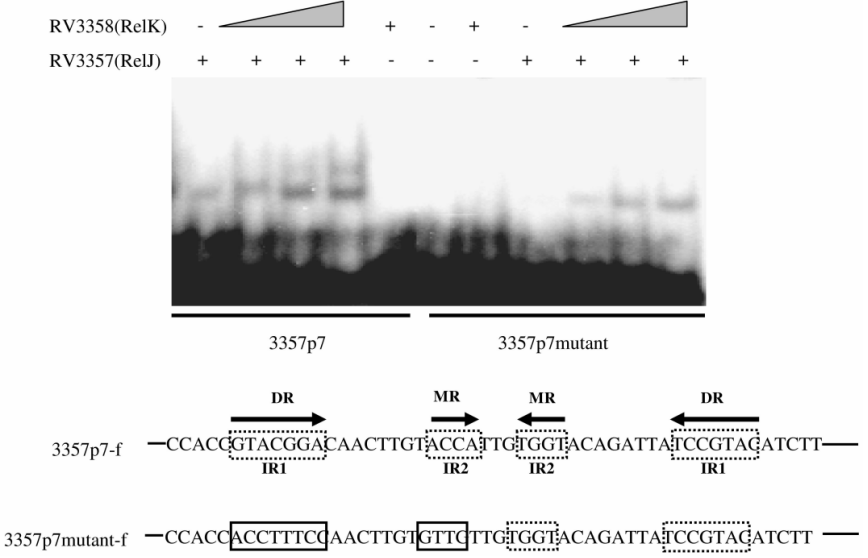
**

Supplement: Figure S2 — EMSA assay for comparing the binding of RelK/RelJ with wild-type and mutant substrates. EMSA and electrophoresis assays were performed as described in the “Materials and Methods”. Wild-type and mutant 3357p7 were used to compare the binding of RelK/RelJ with two substrates. 3357p7 mutant substrates contain mutations within the half of the conserved 3357p7 sequence boxes (DR and MR). The reaction mixtures contain a constant concentration of RelJ (5 µM) and various concentrations of RelK (2.5 µM, 5 µM, and 7.5 µM). (0.13 MB DOC) [file pone.0010672.s005.doc]

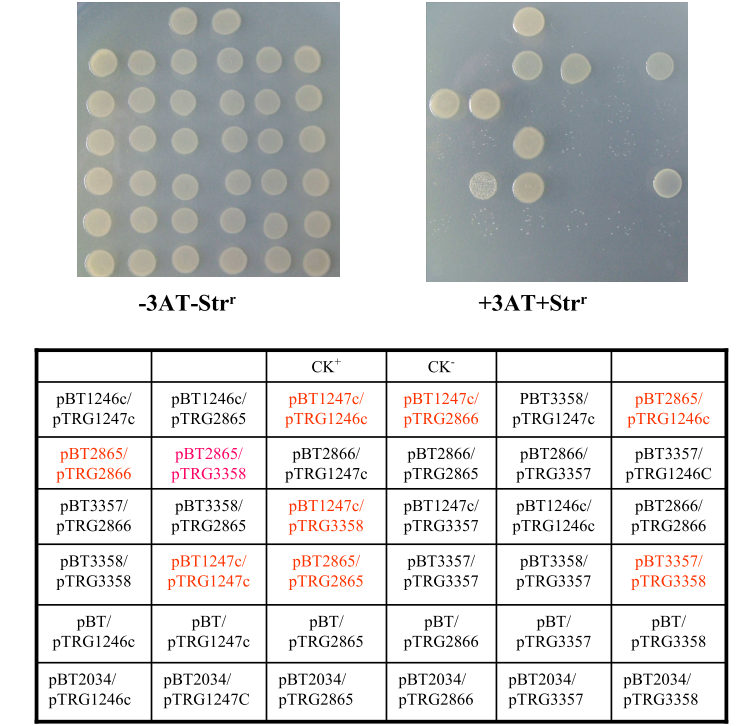

Supplement: Figure S3 — Cross interactions between three pairs of relBE-like genes of M. tuberculosis. The BacterioMatch II two-hybrid system (Stratagene) was used to detect protein-protein interactions of these RelBE protein pairs, as described in the “Materials and Methods”. Up left panel: plate minus streptomycin (str) and 6 mM 3-amino-1, 2, 4-triazole (3-AT). Up right panel: plate plus 10 µg/mL str and 6 mM 3AT. Down panel: an outline of the plates in A, CK+: co-transformant containing pBT-LGF2 and pTRG-Gal11P as a positive control. CK-: co-transformant containing pBT and pTRG as a negative control. Each unit represents the corresponding co-transformant in the plates. All recombinant plasmids and their containing genes were indicated. (0.33 MB DOC) [file pone.0010672.s006.doc]
