# Supplementary material for: Characterization of the Interaction and Cross-Regulation of Three Mycobacterium tuberculosis RelBE Modules
Source: PLoS One. 2010 May 17;5(5):e10672. doi: 10.1371/journal.pone.0010672 (PMC2871789; doi:10.1371/journal.pone.0010672)
Supplement: Supplementary file 5 [file pone.0010672.s007.doc]

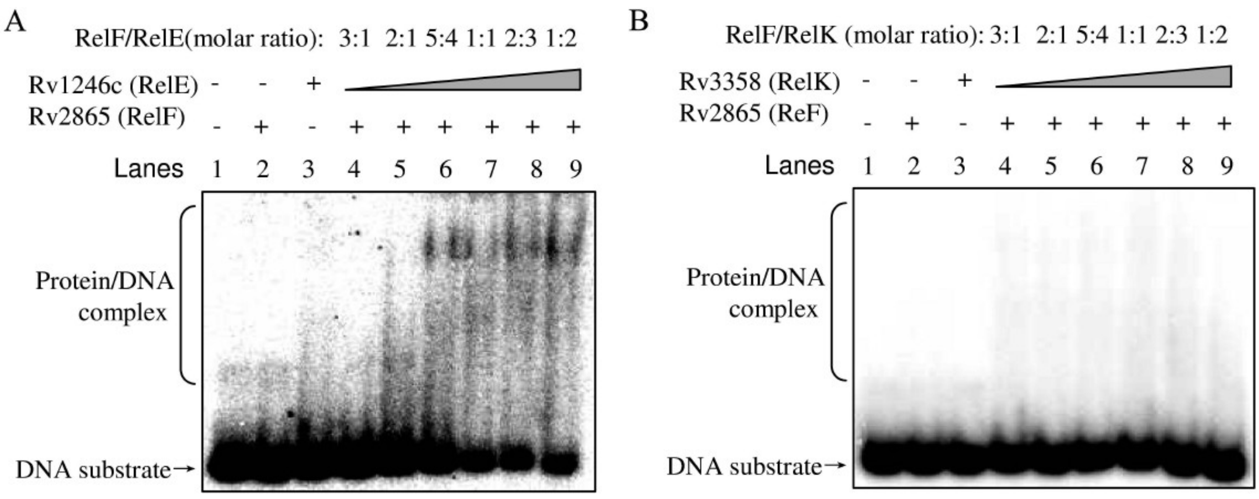

A
RelF/RelE(molar ratio): 3:1 2:1 5:4 1:1 2:3 1:2
Rv1246c (RelE) - - +
Rv2865 (RelF) - + - + + + + + +
Lanes 1 2 3 4 5 6 7 8 9
Protein/DNA complex
DNA substrate
B
RelF/RelK (molar ratio): 3:1 2:1 5:4 1:1 2:3 1:2
Rv3358 (RelK) - - +
Rv2865 (ReF) - + - + + + + + +
Lanes 1 2 3 4 5 6 7 8 9
Protein/DNA complex
DNA substrate
